# Pediatric eye movement-based perimetry: progress, pitfalls, and prospects

**DOI:** 10.3389/fopht.2025.1681070

**Published:** 2025-11-04

**Authors:** Anna Boethun, Sarah Linea von Holstein, René Mathiasen, Miriam Kolko, Frans W. Cornelissen, Jeroen Goossens, Barbara Johanne Thomas Nordhjem

**Affiliations:** 1 Department of Ophthalmology, Copenhagen University Hospital Rigshospitalet-Glostrup, Glostrup, Denmark; 2 Department of Clinical Medicine, Faculty of Health and Medical Sciences, University of Copenhagen, Copenhagen, Denmark; 3 Department of Paediatrics and Adolescent Medicine, Copenhagen University Hospital Rigshospitalet, Copenhagen, Denmark; 4 Department of Drug Design and Pharmacology, Faculty of Health and Medical Sciences, University of Copenhagen, Copenhagen, Denmark; 5 Your Eye Doctors, Eye Hospital Rungsted Kyst, Rungsted Kyst, Denmark; 6 Department of Ophthalmology Aalborg University Hospital, Aalborg, Denmark; 7 Laboratory for Experimental Ophthalmology, University Medical Center Groningen, University of Groningen, Groningen, Netherlands; 8 Department of Cognitive Neuroscience, Donders Institute for Brain Cognition and Behaviour, RadboudUMC, Nijmegen, Netherlands

**Keywords:** perimetry, eye tracking, eye movements, visual fields, visual field tests, pediatrics, child

## Abstract

**Introduction:**

Eye movement-based perimetry (EMP) is a promising, non-invasive approach for visual field assessment, particularly in pediatric populations where standard automated perimetry often fails. However, completion rates in prior pediatric EMP studies have ranged from 41 to 81%, and reasons for unsuccessful testing are seldom reported.

**Objective:**

In this perspective article, we aim to highlight practical barriers and design gaps in EMP systems for children, with a focus on clinical use.

**Observations:**

From our clinical experience with testing two commercially available EMP systems in children (21 patients with brain tumors and 19 age-matched controls), we observed recurring challenges, including poor ergonomic fit, inadequate calibration of eye tracker, and insufficient attention management strategies. These issues frequently led to data loss and incomplete tests, underscoring the gap between current technology and pediatric clinical needs. We outline solutions informed by technological development, vision science and clinical ophthalmology.

**Conclusion:**

Pediatric testing experience must inform EMP design to ensure accessibility and reliability. Our observations highlight the need for clinician-engineer-scientist collaboration, with innovations likely to benefit not only children but also adults with similar testing challenges.

## 1 Introduction

Visual field examination is a cornerstone of diagnosing and managing ophthalmological and neurological disorders. Visual field defects (VFDs) can indicate serious conditions such as retinal disorders, optic neuropathies, or brain tumors (1–3). Early detection and monitoring are essential for timely and appropriate intervention, especially in children (4), where preserving visual function is critical for development and quality of life.

In pediatric patients, reliable visual field assessment is especially important, as some serious conditions initially present through visual complaints (5). For instance, 10-20% of children with brain tumors exhibit VFDs (5, 6) and in some cases this is the only sign of disease. The true prevalence of VFDs in this population is likely higher, but due to limitations in current testing methods, many defects remain undetected (7). This underscores the need for more effective approaches to pediatric visual field assessment.

Traditional perimetric methods, such as Tangent Screen Campimetry, Goldmann Visual Fields (GVF), Frequency Doubling Technology (FDT) and Standard Automated Perimetry (SAP), provide detailed assessments of visual field sensitivity but rely on sustained fixation and manual responses. These cognitive and motor demands make them less suitable for young children. While SAP has been tested in children as young as 5 years old, results are typically unreliable under age 6-7 (8–12), with slightly better outcomes reported only in selected healthy cohorts (13). Notably, none of these studies included children with neurological impairment or visual field defects. Children with these conditions are likely to be at a further disadvantage. Reliability is even lower with other paradigms, with semi-automated kinetic perimetry (Octopus 900) reliable only from around age 11 (14), and FDT from age 8–9 in healthy children (15, 16). GVF, though lacking automated reliability indices, produces acceptable results from age 7–8 (17, 18) and is often preferred under age 9 when compared to SAP and Octopus paradigms (19). Simpler alternatives like confrontational testing are quick and easy to administer but lack sensitivity and rely on examiner skills, limiting their value for follow-up (20).

Eye movement-based perimetry (EMP) offers a promising alternative, leveraging the reflexive saccade: a rapid, automatic eye movement towards a suddenly appearing stimulus in the peripheral visual field (also referred to as visual grasp reflex or exogenous saccade) (21, 22). This instinctive gaze shift is conceptually similar to the response observed in preferential looking paradigms, widely used to assess visual function in infants and toddlers. In some EMP systems, the saccadic movement simply substitutes the manual response (23), while others quantify saccadic reaction time (SRT) - the latency from stimulus onset to saccade initiation - as a proxy for visual field sensitivity (24, 25). In adult cohorts, EMP has demonstrated feasibility and diagnostic value in glaucoma as well as various neurological disorders (23, 26, 27). EMP has also been tested in both healthy and visually impaired children, with varying degrees of success (28–34). Across pediatric EMP studies, a consistently high proportion of children, ranging from 41% to 81%, remain partially tested or untested.

Numerous EMP devices are currently under development, most targeting glaucomatous visual field loss in adults. Prior studies have shown that these methods are more intuitive and easier for adults to perform than traditional SAP (23, 35). Our objective was to determine whether systems originally developed for adults (adult systems) can also be used to examine children. We selected two European eye-tracking systems for evaluation in 4-18-year-olds: BulbiCAM (36) (BulbiTech AS, Trondheim, Norway), which mimics SAP with a static-grid paradigm, and SONDA (37) (the Standardized Oculomotor Neuro-ophthalmic Disorders Assessment; Reyedar, Groningen, The Netherlands), which relies on continuous tracking of a moving target.

Translating EMP to pediatric use introduces unique challenges: children’s limited comprehension and attention, physical constraints, and immature visual and oculomotor systems complicate direct application of adult setups. In this perspective article, we describe pitfalls and usability issues when testing BulbiCAM and SONDA in a pediatric cohort. We present practical lessons and recommendations to support improved EMP implementation for children.

## 2 Description of two adult systems

**Figure 1:**
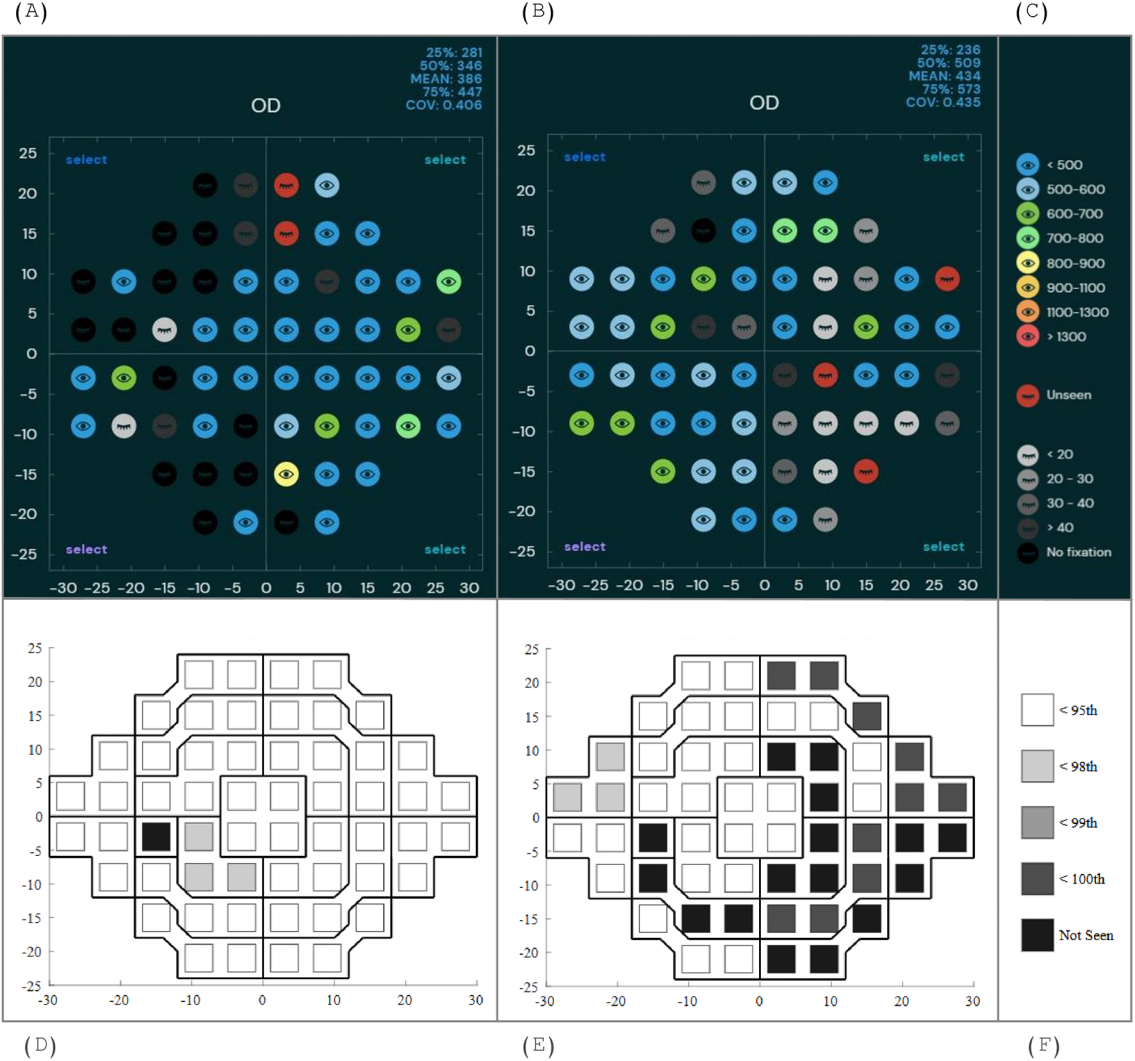
Visual field plots from one patient (9-year-old with temporal hemianopia) and one age-matched control. **(A)**. healthy control tested with BulbiCAM, **(B)**. patient tested with BulbiCAM, **(C)**. labels, numbers ranging from 500 to 1300 indicate saccadic reaction time in ms, **(D)**. healthy control (SONDA), **(E)**. patient (SONDA), **(F)**. labels, numbers indicate SRT-percentile.

The technical specifications and EMP paradigms for BulbiCAM and SONDA are provided in **Table 1**, including mounting options, calibration requirements, stimulus properties, and the criteria for visual field assessment. The standard output of BulbiCAM and SONDA is shown in **Figure 1**.

**Table 1:** Technical specification and description of the systems and visual field tests for BulbiCAM and SONDA.

Info	BulbiCAM	SONDA
System Type	Haploscope with integrated eye tracker and +6 lenses placed between eye and virtual image; Software run via accompanying PC	Standardized oculomotor assessment platform (monitor, wearable tracker, companion device (smart phone) and accompanying PC)
Eye Tracker Specs	Glint based; 400 Hz (alternating 200 Hz bright pupil/200 Hz dark pupil glint); effective 200 Hz	Video-based; 120 Hz; head-mounted (Pupil Labs Pupil Invisible)
Monitor specs	14.7 cm viewing distance, screen dimensions: 121x68 mm, 60 Hz monitor refresh rate	60 cm viewing distance, screen dimensions: 598x336 mm, 60 Hz monitor refresh rate
Mounting Options	Ceiling or desk mount (desk-mounted used in study)	Monitor on height-adjustable table with chinrest
Calibration	None required (fixation assumed to align with target at start of each trial)	Calibration-free (deep learning-based analysis); uses screen detection for head movement correction
Fixation Target	Green, moves smoothly 4 times (center: all corners) to sample different field locations, present at all times	See “stimulus details”
Stimulus Details	White stimuli, Goldmann III4e equivalent, on gray (10 cd/m²) background; three intensity onsets (instant, logarithmic, linear; instant used)	Moving Gaussian blob (0.43°, Goldmann III, peak 42 cd/m²) on gray background (30 cd/m²)
Visual Field Protocol	60 points per eye, covering central ±30° (standard protocol).	56 points peer eye (24–2 standard pattern + two points) via smooth pursuit and step/jump to new locations.
Testing time	2 to 4 minutes per eye (depending on performance).	4x40 seconds per eye.
Participant Instructions	“Look at the green dot, when you see a white dot look at that and then look back at the green dot”	“Follow the white dot with your eyes”
Requirements to show stimulus	Gaze within a 5-pixel radius circle (eye coordinates) for ≥300 ms within 3 seconds prior to stimulus onset; if not met, stimulus not displayed and position marked as “no fixation” (black).	None
Definition ‘Seen’/’Unseen’	“Seen” if gaze moves ±30° toward stimulus 120–1200 ms after onset and subsequent fixation detected (63 ms, gaze within 1.5 px radius circle); otherwise “unseen” (red in plot) or by distance measure (Euclidean distance from target-stimulus vector; gray scale in plot)	“Seen” if saccade (gaze velocity 30 deg/s or gaze acceleration 175 deg/s^2^) starts within 8° of pre-jump stimulus position and ends within 8° of post-jump stimulus position; otherwise “Unseen”
Response Time Mapping	Color coding based on latency (time to leave fixation area after stimulus onset)	Grayscale coding by SRT quartiles compared to adult, normative data
Retesting	Locations marked as “no fixation” or “unseen” retested once	None
Manual Review	Examiner review and exclude falsely categorized “seen” responses	None
Other Analysis	None	Machine learning based analysis of Spatiotemporal properties (not performed in current study due to small sample size)

## 3 Clinical experience and testing context

Our experience using BulbiCAM and SONDA is informed by EMP testing in 21 patients aged 4–18 years diagnosed with a brain tumor and 19 age-matched healthy controls. Patients were recruited from the Department of Ophthalmology, Rigshospitalet, Copenhagen, between March 15, 2023, and September 31, 2024, following referral by the treating pediatric ophthalmologist. Healthy controls were recruited through the authors’ personal networks and were excluded if they had a history of ophthalmic/neurologic disease or abnormal findings upon ophthalmic examination. The EMP testing was conducted in a dimly lit examination room at the Department of Ophthalmology, Rigshospitalet, Copenhagen. All examinations were conducted by staff experienced in pediatric ophthalmic testing.

The BulbiCAM was placed on a height-adjustable table and adapted to each child, allowing for either seated or standing testing. Bulbitech provided custom face pieces for smaller head sizes, along with a stable mounting base. These face pieces were secured to the child’s head using an elastic band and then magnetically attached to the haploscope to reduce head movements. The test was operated through a user-interface (BulbiHUB).

The SONDA setup comprised a monitor mounted on a height-adjustable table with an attached chinrest. If necessary, the child’s head was gently held in place by the examiner to minimize movement. Children were seated in a height-adjustable chair. The eye tracker was connected to a mobile device, which was linked to a laptop. The SONDA test was operated through the Ubuntu terminal.

## 4 Main issues encountered

EMP in children presents age-specific challenges across hardware, testing procedures, and data interpretation. Below, we outline the main problem areas in four sections:

### 4.1 Hardware and ergonomics

#### 4.1.1 Eye tracker hardware

Most eye-tracking equipment is designed with adult users in mind, which poses challenges in pediatric applications. The head-mounted eye tracker used with SONDA was only available in an adult-size at the time of testing, resulting in slippage, despite the use of two elastic bands for stabilization. Reyedar now offers a child-size wearable eye tracker (Pupil Labs GmbH Neon) for use with SONDA.

Similarly, BulbiCAM requires a head strap to minimize movement, but younger children found this setup distressing. As a result, testing in these cases was conducted without the head strap, which may have allowed minor head movements during data collection.

#### 4.1.2 Seating and mobility

Room furnishings must accommodate all body sizes and mobility needs. The standard ophthalmic chin rest used with the SONDA setup was too tall for smaller children, increasing head movements due to poor headband reach. Similarly, tables and chairs are often too large and not easily adjustable or movable for wheelchair users. Ensuring that children feel comfortable and safe during testing is essential, which includes offering the choice to remain in their own wheelchair or sit on a parent’s lap. In one case, a wheelchair-using child was unable to get close enough to the screen for reliable eye tracking. Tschopp et al. (8) addressed these challenges by using a custom pediatric chair.

### 4.2 Tracking and calibration

#### 4.2.1 Pupil detection

In our initial BulbiCAM trials, many children’s eyes were untrackable. Similar issues have previously been described in the literature and might be explained by watery eyes and large pupils that complicate glint and pupil detection, leading to intermittent data loss (38, 39). Tobii (another eye tracking manufacturer) even launched a specific illumination mode specifically designed for tracking infants’ gaze (40). We found that reducing the IR diode output solved the issue, although this is not a user-accessible setting.

#### 4.2.2 Algorithmic bias

Video-oculography systems like the Pupil Labs Invisible used with SONDA rely on machine learning algorithms trained exclusively on healthy adult data (41, 42). This may introduce inaccuracies in pediatric as well as clinical populations. Although gaze estimates appeared broadly plausible, formal pediatric validation is needed. Retraining the algorithms on pediatric data could further improve accuracy.

#### 4.2.3 Calibration trade-offs

Traditional multi-point calibration requires comprehension, cooperation, and sustained attention, which is often challenging in children (28, 32). Both SONDA and BulbiCAM sidestep this procedure: SONDA uses the calibration-free Pupil Invisible eye tracker, while BulbiCAM uses an uncalibrated eye tracker in combination with task adaptations and assumptions about fixation position. The benefit is speed - but at the cost of potentially imprecise or even inaccurate gaze estimations, limiting accurate localization of responses within the visual field.

### 4.3 Stimulus design and attention management

#### 4.3.1 Test duration and breaks

Incomplete tests were common in our cohort and have also been reported in other pediatric visual field studies (8, 28), highlighting the need for pause options in this population. Both systems used in our study lack pause buttons to accommodate breaks, which is a limitation given children’s limited attention spans.

SONDA partially mitigates this issue by dividing the test into four trials, each lasting ~40 seconds, thereby allowing for three short breaks per eye. However, this structure has a critical limitation: interrupted trials or incomplete tests cannot be analyzed, which reduces its robustness in less cooperative or fatigued participants.

BulbiCAM requires a single uninterrupted session per eye. Although this increases the demand on the participant, unfinished tests can still be analyzed, providing greater flexibility. Additionally, Bulbitech offers shorter paradigms with 16 or 26 test points. However, the current design tests one quadrant in its entirety before proceeding to the next, which increases the risk of a quadrant being affected by temporary inattention. If a pause function was added, the sequential structure could potentially improve the reliability of defect detection by maintaining localized testing within manageable time blocks.

#### 4.3.2 Engagement strategies, test and stimulus characteristics

Many children found the tests boring. Some even struggled to stay awake. The dynamic fixation target used in SONDA seemed to have a slight advantage in keeping children engaged. Nonetheless, active encouragement from the examiner was required to sustain attention throughout the test - especially when testing patients and younger children.

When tested with BulbiCAM, children quickly recognized the predictable sequence in which stimuli were presented (each eccentricity tested from top to bottom before progressing) which made the test easy to anticipate and, consequently, less engaging. For children with VFDs, locating the fixation target after a saccade was often challenging, leading to search behavior.

### 4.4 Developmental aspects

#### 4.4.1 Normative/ground-truth

Children’s oculomotor and visual systems mature during the first years of life (43, 44). Therefore, pediatric normative datasets spanning a wide age range are crucial to capture this developmental change. However, such datasets for comparison and training purposes are currently missing. In addition, conventional methods such as SAP or GVF are unreliable in young children and cannot be easily used for comparison, highlighted by the fact that some EMP studies lack any comparative test altogether (30, 33).

#### 4.4.2 Saccadic reaction time

Both BulbiCAM and SONDA use SRT as a proxy for visual field sensitivity, enabling rapid data collection. However, SRT is strongly influenced by factors such as stimulus contrast, cognitive load, and age (45, 46). Given these dependencies, its suitability as a reliable proxy is questionable, particularly in children with delayed cognitive development or neurological conditions affecting oculomotor control and attention.

#### 4.4.3 Fixation behavior

The BulbiCAM paradigm requires stable fixation to present stimuli, but in younger children and those with VFDs, this often failed due to inattention or poor tracking. As a result, fewer stimuli were shown, reducing engagement further, and causing interruptions. Additionally, children’s fixation behavior differs from that of adults (47), making standard assumptions about gaze events less applicable in pediatric testing.

#### 4.4.4 Data quality and noise management

Inspection of raw data from our trials revealed that gaze often did not follow the stimulus sequence or trajectory as expected. It was difficult to determine whether this was due to measurement noise, poor performance, or true visual field loss. Accurately distinguishing between these sources of error is essential for interpreting results and assessing the measurement reliability.

## 5 The way forward: towards child-centered EMP design

Building on our experience with BulbiCAM and SONDA, as well as insights from the broader literature, we offer recommendations to guide further development of EMP for children. We propose child-friendly solutions organized around the four previously mentioned problem areas:

### 5.1 Prioritize child-specific ergonomics and reliability

Our testing was marked by limited adaptability of setup and recurring hardware and software failures. Test setups must accommodate children while maintaining the robustness essential for clinical use:

#### 5.1.1 Design with the child in mind

Hardware should accommodate children’s size and movement while reliably capturing raw data. Wearable options, such as virtual reality (VR) headsets, may improve stability and allow natural head movements, potentially making the test more comfortable and engaging. VR has shown promise for static perimetry in children as young as nine (48), though further research into usability in younger children and EMP specifically is needed. For non-wearable systems, test setups should be equipped with adjustable tables, seating and mounting solutions to optimize test conditions for all children, including wheelchair-users.

#### 5.1.2 Robust systems

Unlike adult participants, who may tolerate occasional equipment malfunctions or repeated trials, children have minimal tolerance for such disruptions. Therefore, systems intended for clinical use should undergo especially rigorous testing for hardware and software reliability, as system robustness is critical even during early-stage trials in pediatric populations.

### 5.2 Refine tracking and calibration techniques for pediatric use

A major lesson from current systems is the necessity for adaptive, user-friendly tracking and calibration methods, explicitly validated in pediatric populations:

#### 5.2.1 Illumination modes

Glint based eye tracking systems should offer automated and user-accessible IR level controls or illumination mode settings, allowing for tuning to each individual variations in pupil size and iris reflectivity commonly encountered in children.

#### 5.2.2 Pediatric-trained algorithms

Future machine learning models for gaze and event detection, as well as data analysis, must be trained on data from children across multiple developmental stages. Age-stratified datasets are essential for capturing a full range of typical developmental variability, pupil characteristics, and oculomotor behaviors seen in the pediatric population.

#### 5.2.3 Practical calibration methods

Calibration procedures should balance ease-of-use with spatial accuracy. Promising approaches include calibration-free eye trackers (e.g., Pupil Labs Neon), smooth pursuit calibration, single-point systems (Tobii Pro Glasses 3), and stereoscopic setups using two cameras per eye (49). However, combining uncalibrated eye trackers with test compliance requirements (e.g., fixation requirements) should be avoided, as this results in poor spatial resolution and likely increases overall testing time in the children.

### 5.3 Attention through stimulus and engagement design

Maintaining attention and ensuring accessibility for all children, regardless of impairment, requires thoughtful stimulus and paradigm design. Several promising strategies include:

#### 5.3.1 Easy pause-and-resume

Tests must include simple, pause and resume functions, allowing children breaks without losing data or the need for restarting entire test segments.

#### 5.3.2 Dynamic fixation and stimuli

Based on our experience, children find it more natural to follow a moving target with their gaze, which in turn enhances engagement. We therefore suggest that future paradigms prioritize dynamic over static stimuli. BulbiCAM offers an optional “green dot animation” feature (a red circle contracting towards the fixation point) to help guide gaze. This feature may be useful but was not employed in order to maximize comparability to other tests and studies.

#### 5.3.3 Color and contrast accessibility

Stimuli should be standardized in terms of luminance contrast, while additional gaze-guiding features - such as the green dot animation used in BulbiCAM - may incorporate color. However, such elements should be designed with common forms of color vision deficiency in mind to ensure visibility and usability for all children, regardless of their color perception.

#### 5.3.4 Gamification and natural viewing

Simple gamification is feasible in certain pediatric age groups (4–12 years) (50). However, the cognitive demands of such designs must be carefully considered to avoid excluding younger or cognitively impaired children. A more universally accessible approach may involve attention-capturing stimuli, such as movie clips, as demonstrated by Allen et al. (51). Additionally, gaze-contingent paradigms and free-viewing of naturalistic scenes, which have been explored in adult populations (52, 53), may offer promising directions for developing more child-friendly EMP tests in the future.

### 5.4 Toward reliable and interpretable pediatric EMP data

#### 5.4.1 Age-stratified normative databases

The absence of comprehensive normative pediatric datasets poses a major limitation for both clinical interpretation and research. Such reference data describe what is typical at different developmental stages, including age-related changes in eye movement patterns, fixation stability, and saccadic reaction time. These norms are essential not only for identifying deviations that may signal pathology, but also for guiding model development and validation.

#### 5.4.2 Thresholding and saccadic reaction time analysis

The suitability of SRT as a proxy for visual field sensitivity requires further investigation - particularly in children - before it can be considered reliable. An alternative approach may lie in the gradual-onset stimulus mode available in BulbiCAM, where the stimulus progressively increases in luminance. In such cases, SRT becomes less meaningful, and the luminance level that triggers the saccade should be recorded instead. However, this method would likely increase both testing and waiting time, which may limit its feasibility in pediatric populations.

#### 5.4.3 Novel reliability indices for EMP in children

Standard automated perimetry (SAP) provides easy-to-interpret reliability indices based on fixation losses, false positives, and false negatives. Comparable metrics are currently lacking in EMP, but should be developed. These could reflect the number of incorrectly presented stimuli, missing raw data, and saccade characteristics such as accuracy and precision. Age-adjusted scoring would be essential to account for the immaturity of the oculomotor system in younger children.

## 6 Discussion

This perspective paper, based on experience evaluating two commercial EMP systems in a diverse pediatric sample, highlights multifaceted challenges related to hardware ergonomics, eye tracking and calibration, stimulus engagement, and developmental considerations. To our knowledge, pediatric testing experiences with EMP systems have not been systematically described in the literature.

Research on EMP in children is limited, with only three systems tested: SVOP (28–32), EMPP (34), and “Field Bubbles” (33). All incorporated child-friendly features but relied on calibration, which failed in 5–25% of cases - except “Field Bubbles”, which successfully used a one-point calibration procedure. Overall, successful completion rates in pediatric EMP studies are low (41–81%) (28–34). Direct comparison between studies is limited by differences in methodology, reporting, study populations, and the absence of normative data or reference standards. Together with our observations, this underscores the need for coordinated progress in pediatric EMP development.

Several additional EMP systems are currently under development and being tested in adults (23, 52, 54, 55). It is likely that these systems will soon be tested in children as well, especially given that a recent systematic review identified EMP as a promising emerging technology for pediatric visual field testing (56). To avoid repeating earlier pitfalls in system development, set-up, and study design, we believe that our observations can provide valuable guidance for future pediatric EMP research and development.

## 7 Limitations

Our perspective is grounded in hands-on clinical experience with two adult EMP systems adapted for use in children. To our knowledge, this is the first report directly comparing multiple systems in a pediatric setting, but it does not capture the full diversity of emerging EMP approaches. The population tested ranged from 4–18 years of age, which limits our ability to comment specifically on the feasibility of EMP in very young children. Our recommendations are intentionally broad and not system-specific, reflecting the early stage of EMP development in children and the absence of established standards. While principles such as ergonomic flexibility, attention-capturing stimuli, and simplified calibration may appear self-evident, these aspects have not yet been formalized into design guidelines and are not consistently addressed in existing systems.

## 8 Conclusion

Incorporating pediatric testing experience into EMP design is essential to ensure accessibility, reliability, and clinical value. Our observations underscore the importance of interdisciplinary collaboration - particularly between clinicians, engineers, and vision scientists - to adapt these tools for pediatric use. While our focus is on children, such innovations may also inform adaptations for adult populations with comparable cognitive or physical challenges.

## Data Availability

The raw data supporting the current study are not publicly available due to data protection reasons but are available from the corresponding author on reasonable request.
